# Association between intraoperative balloon morphology and postoperative sensory dysfunction after percutaneous balloon compression for trigeminal neuralgia: a propensity score–matched cohort study

**DOI:** 10.3389/fneur.2026.1918125

**Published:** 2026-09-24

**Authors:** Bin Zhu, Boyang Yan, Dongmei Xu, Jinfeng Pang, Haoyu Dong, Xianbin Ning

**Affiliations:** 1 Department of Neurosurgery, Affiliated Hospital of Beihua University, Jilin, China; 2 Medical Records Room, The Central Hospital of Jiamusi City, Jiamusi, China

**Keywords:** balloon morphology, percutaneous balloon compression, propensity score matching, sensory dysfunction, trigeminal neuralgia

## Abstract

**Background:**

Percutaneous balloon compression (PBC) is widely used for trigeminal neuralgia, but the association between intraoperative balloon morphology and postoperative sensory outcomes remains unclear. Defining this relationship may improve technique and risk stratification. Prior studies suggest selective injury to large myelinated fibers rather than generalized sensory fiber or ganglion injury, and balloon morphology should be interpreted as a procedural marker rather than a direct measure of neural injury.

**Methods:**

We retrospectively identified patients who underwent PBC for trigeminal neuralgia from institutional records. Of 603 screened patients, 566 met eligibility criteria. Sensory outcomes were assessed using standardized examinations and a modified Barrow Neurological Institute (BNI) classification. The primary endpoint was moderate-to-severe sensory dysfunction at 3 months. Propensity scores based on demographic, clinical, and biochemical variables generated 102 matched pairs. Univariable and multivariable logistic regression evaluated the association between balloon morphology and postoperative sensory dysfunction, with prespecified subgroup analyses by disease duration and procedural factors. Balloon morphology was analyzed as a fluoroscopic procedural marker; intraluminal pressure was not measured.

**Results:**

Among 566 eligible patients, 118 (20.8%) developed moderate-to-severe postoperative sensory dysfunction, while 448 had none or mild impairment. After matching, 204 patients were analyzed, and baseline covariates were well balanced (standardized mean differences <0.1). Non–pear-shaped balloon configuration was more frequent in patients with moderate-to-severe sensory dysfunction. In multivariable analysis, non–pear-shaped morphology remained associated with higher odds of moderate-to-severe impairment at 3 months (adjusted OR, 1.82; 95% CI, 1.09–3.05; *p* = 0.021), though this reflects an observational association rather than causal injury. Longer disease duration and smaller balloon volume were also significant predictors, whereas compression time showed a weaker, statistically uncertain association. Subgroup analyses showed consistent risk elevations for non–pear-shaped morphology without significant effect modification.

**Conclusion:**

Intraoperative balloon morphology is observationally associated with sensory outcomes after PBC. Non–pear-shaped patterns may indicate higher risk of moderate-to-severe sensory dysfunction and serve as a procedural quality marker, but this should not be interpreted as direct ganglion injury or generalized sensory fiber damage.

## 1 Introduction

Trigeminal neuralgia is characterized by sudden, intense facial pain and remains one of the most debilitating craniofacial conditions encountered in clinical practice (1, 2). Patients frequently rely on sodium channel blockers for symptom control (3–5), yet pharmacological responsiveness diminishes over time and adverse effects limit sustained use (6). As a result, many individuals require interventional treatment (7–9). Percutaneous balloon compression has emerged as a widely adopted option because it provides rapid pain relief and can be administered with a favorable safety profile (10–12). Although the procedure is effective in interrupting nociceptive transmission, the exact mechanisms leading to postoperative sensory outcomes remain incompletely understood (13). Prior studies have demonstrated that balloon morphology is associated with postoperative sensory dysfunction (14), but many of these reports did not rigorously account for baseline clinical and demographic confounders. Experimental and anatomical studies suggest that standard balloon inflation within Meckel’s cave primarily occupies the trigeminal cistern and elevates the dural layers surrounding the ganglion, rather than directly deforming trigeminal ganglion cell bodies (10, 15). Intraoperative fluoroscopy frequently demonstrates characteristic balloon morphologies, which serve as imaging markers of balloon positioning and expansion rather than indicators of direct ganglion compression. A pear-shaped balloon configuration has traditionally been regarded as an indicator of appropriate catheter placement and balloon expansion within Meckel’s cave, whereas non–pear-shaped configurations may reflect anatomical variation or technical factors affecting balloon positioning (16–18). Deviations from this configuration, which can arise from individual anatomical differences or technical factors, have been linked in prior studies to differences in analgesic durability and sensory outcomes (19). The observed association between smaller filling volumes and increased sensory impairment likely reflects balloon interaction with the proximal retroganglionic fibers near the porus trigeminus rather than compression of ganglion cell bodies (20).

Standardized tools such as the Barrow Neurological Institute facial numbness scale provide a structured framework for evaluating sensory outcomes and allow classification of postoperative numbness across a gradient of severity (21, 22). Moderate and severe numbness are particularly relevant end points because they reflect deficits that are persistent and functionally meaningful (23). Understanding which procedural features influence the likelihood of developing these deficits is essential for refining operative strategy and counseling patients regarding expected outcomes.

To provide robust evidence within a well-characterized cohort, the present study evaluates the association between intraoperative balloon morphology and postoperative sensory dysfunction in a large population of patients treated for trigeminal neuralgia. Unlike prior studies, a propensity score matching approach was applied to balance baseline factors, enabling more reliable comparison of sensory outcomes. Importantly, balloon morphology is evaluated as an imaging marker of procedural configuration rather than a direct surrogate for ganglion compression or intraluminal balloon pressure, neither of which was measured in this study.

## 2 Methods

### 2.1 Study design and setting

This retrospective cohort study included consecutive patients with trigeminal neuralgia who underwent percutaneous balloon compression (PBC) at our tertiary-care, municipal referral neurosurgical center between January 2019 and December 2024. The center routinely performs PBC for patients with medically refractory trigeminal neuralgia, and clinical records generated during this period were systematically reviewed to identify eligible patients. Trigeminal neuralgia was diagnosed according to the International Classification of Headache Disorders, 3rd edition (ICHD-3) criteria. Patients with secondary trigeminal neuralgia caused by identifiable structural lesions, multiple sclerosis-associated trigeminal neuralgia, or other neurological disorders were excluded to maintain a homogeneous cohort of patients with primary trigeminal neuralgia undergoing PBC. The study cohort consisted of unique patients rather than individual procedures. Each patient contributed only one eligible percutaneous balloon compression procedure to the analysis. For patients who underwent repeat PBC during the study period, only the first eligible procedure was included to avoid multiple observations from the same individual and to ensure independence of observations in the statistical analyses.

A total of 603 patients who underwent PBC during the study period were initially identified. Patients were excluded if they had incomplete clinical or procedural data, inadequate intraoperative fluoroscopic images for balloon morphology assessment, previous destructive trigeminal procedures (such as radiofrequency thermocoagulation, glycerol rhizotomy, or other ablative interventions), or insufficient postoperative follow-up for evaluation of sensory outcomes. After applying the exclusion criteria, 566 patients were included in the final analysis.

Clinical characteristics, procedural parameters, intraoperative balloon morphology, and postoperative sensory outcomes were collected from electronic medical records and operative reports. The primary exposure variable was intraoperative balloon morphology, classified as pear-shaped or non–pear-shaped configuration based on fluoroscopic assessment. The primary outcome was moderate-to-severe postoperative sensory dysfunction during follow-up.

### 2.2 Patient selection and eligibility criteria

A comprehensive review of institutional records identified a total of 603 patients who underwent percutaneous balloon compression for trigeminal neuralgia during the defined study interval. From this source population, eligibility was determined through a sequential screening process intended to ensure diagnostic precision and adequate documentation of perioperative and follow-up parameters. Individuals were first required to meet clinical diagnostic standards compatible with internationally accepted criteria for trigeminal neuralgia, including paroxysmal, distribution-specific facial pain and the absence of alternative neuropathic conditions on clinical assessment or neuroimaging (24–26). Patients in whom diagnostic ambiguity persisted or in whom secondary causes could not be reasonably excluded were removed at this stage.

Further exclusions were necessary to maintain the integrity of outcome evaluation. Cases lacking complete operative documentation, intraoperative fluoroscopic imaging, or postoperative sensory assessments at the predetermined intervals of 1, 3, and 12 months were not retained. Patients who had undergone prior ablative trigeminal interventions other than microvascular decompression (MVD) were similarly excluded to avoid confounding influences on postoperative sensory outcomes. After these criteria were applied, 566 patients remained available for analysis. These 566 eligible patients constituted the source cohort for the exposure-based propensity score analysis, in which patients with non–pear-shaped balloon configurations were subsequently matched to those with pear-shaped balloon configurations.

### 2.3 Percutaneous balloon compression procedure

Percutaneous balloon compression was performed under general anesthesia using fluoroscopic guidance with standard needle placement through the foramen ovale into Meckel’s cave. Written informed consent for the clinical PBC procedure was obtained from all patients before surgery. All procedures were performed by four neurosurgeons experienced in percutaneous trigeminal procedures during the study period. A standardized operative protocol was applied throughout the study period, including fluoroscopic-guided foramen ovale access, balloon catheter placement, contrast-assisted balloon inflation, standardized assessment of balloon morphology, and consistent postoperative sensory evaluation. A No. 4 Fogarty balloon catheter was introduced through the cannula and advanced toward the porus trigeminus. Incremental instillation of nonionic contrast medium allowed controlled balloon inflation, and fluoroscopic images were obtained to document balloon configuration. Balloon inflation was maintained for a standardized duration, followed by catheter deflation and removal. Balloon volume and compression duration were recorded as procedural parameters.

Balloon morphology, considered the primary exposure variable, was assessed using intraoperative fluoroscopic images obtained at maximal balloon inflation after complete filling with contrast medium. Images were acquired using biplanar fluoroscopy, including anteroposterior (AP) and lateral projections, to evaluate balloon contour and anatomical positioning. Two board-certified neurosurgeons independently reviewed the fluoroscopic images while blinded to postoperative sensory outcomes and to each other’s assessments. Based on balloon contour and anatomical positioning, balloon configurations were classified into five categories: pear-shaped, oval-shaped, dumbbell-shaped, irregular-shaped, and poorly positioned balloons. Representative fluoroscopic examples of each morphology subtype are shown in Supplementary Figure S1. The Pear-shaped morphology was defined as a balloon demonstrating a characteristic waist at the level of the trigeminal cistern/Meckel’s cave. Oval-shaped balloons were defined as symmetric expansion without a distinct waist, dumbbell-shaped balloons as two-lobed configurations caused by uneven expansion, irregular-shaped balloons as asymmetric or distorted contours, and poorly positioned balloons as those with inadequate anatomical positioning within the intended compression region.

In cases of disagreement between the two assessors, the fluoroscopic images were re-evaluated jointly, and a consensus classification was reached through discussion. Borderline cases in which balloon morphology could not be clearly assigned to a single category were reviewed based on both balloon contour and anatomical positioning using the available biplanar fluoroscopic views. If consensus could not be achieved, the final classification was determined by a third senior neurosurgeon with expertise in percutaneous trigeminal procedures. For the primary analysis, balloon morphology was dichotomized into pear-shaped and non–pear-shaped configurations, with all atypical morphologies combined into the non–pear-shaped group. Interrater reliability of balloon morphology classification was assessed using Cohen’s κ, demonstrating high agreement between observers (κ = 0.85; 95% CI: 0.78–0.92).

### 2.4 Assessment of sensory outcomes

Postoperative sensory evaluations were performed using a structured protocol that incorporated both clinical examination and patient-reported symptoms. BNI facial numbness scale was adopted as the primary instrument for quantifying sensory change. This five-level scale characterizes postoperative sensation as entirely normal in grade I, mildly altered but not functionally limiting in grade II, clearly reduced yet tolerable in grade III, distinctly bothersome in grade IV, and severely disruptive in grade V. Examiners assigned a grade at each follow-up visit based on findings from targeted sensory testing and the patient’s qualitative description of facial perception. Clinic-based follow-up was complemented by telephone interviews when necessary.

For analytical purposes, the ordinal structure of the BNI scale was reorganized into four clinically meaningful categories. Patients classified as grade I were categorized as having no sensory impairment. Grade II was interpreted as mild impairment. Grades III and IV were combined to represent moderate impairment, reflecting the presence of clear sensory loss with measurable influence on daily activities. Grade V was designated as severe impairment, indicating substantial and persistent sensory disruption. The principal outcome of this study was defined as the presence of moderate or severe impairment at the three-month follow-up visit, which was selected because postoperative sensations typically stabilize during this interval.

Sensory assessments were performed at 1, 3, and 12 months after the procedure by clinicians trained in neurologic evaluation. Each examination incorporated standardized testing of tactile discrimination, light touch, and proprioceptive awareness within the affected trigeminal divisions. When the patient’s subjective description diverged from the objective findings, a final classification was assigned through a documented clinical judgment that integrated both perspectives. All assessments were reviewed for completeness before inclusion in the analytic cohort to ensure reliable characterization of postoperative sensory outcomes.

### 2.5 Covariates and data collection

Clinical, procedural, and physiological data were abstracted from the institutional electronic medical record using a structured extraction framework designed to maintain uniformity across all observations. Variables were selected *a priori* based on their established or plausible relevance to postoperative sensory outcomes in trigeminal neuralgia. Demographic characteristics included patient age, sex, and the side of symptom involvement. Disease-specific information encompassed the duration of trigeminal neuralgia prior to intervention, the distribution of pain across the trigeminal divisions, and the presence of recurrent or refractory symptoms. These elements were obtained from standardized neurological evaluations conducted during preoperative consultations.

To account for comorbid conditions that might influence neural vulnerability or postoperative recovery, data related to hypertension, diabetes mellitus, and other systemic illnesses were collected from medical histories and verified through medication lists and laboratory records. Physiological and biochemical parameters, including systolic blood pressure, fasting glucose concentration, and serum alanine aminotransferase, were retrieved from perioperative laboratory assessments performed within 24 h of the procedure. These variables were treated as continuous measures unless clinical convention supported categorical interpretation. All covariates were extracted from routine preoperative assessments and standard laboratory panels. Operative characteristics, such as balloon volume and compression duration, were derived from anesthetic and surgical documentation completed contemporaneously during the intervention.

All extracted data underwent quality review before entry into the analytic dataset. Discrepancies were adjudicated by an investigator not involved in outcome assessment to ensure that misclassification did not arise from transcription or interpretation errors. Missing values were infrequent and, when present, were resolved through verification of the original clinical record. The assembled dataset provided a comprehensive set of covariates necessary for propensity score modeling and for subsequent regression analyses examining the association between procedural features and sensory outcomes.

### 2.6 Propensity score development and matching

Because balloon morphology was the primary exposure of interest, propensity scores were estimated as each patient’s probability of having a non–pear-shaped rather than a pear-shaped balloon configuration. The propensity score model included prespecified demographic, disease-related, comorbidity, physiological, previous treatment-related, procedural, and surgeon-related variables that could plausibly be associated with balloon configuration and postoperative sensory outcomes. Postoperative sensory outcomes were not included in propensity score estimation.

Patients with non–pear-shaped balloon configurations were matched 1:1 to patients with pear-shaped configurations using nearest-neighbor matching without replacement and a caliper width of 0.2 standard deviations of the logit of the propensity score. Covariate balance before and after matching was evaluated using standardized mean differences (SMDs), with an absolute SMD < 0.10 considered indicative of adequate balance. The resulting propensity score–matched cohort was used for the primary analysis of the association between intraoperative balloon morphology and moderate-to-severe sensory dysfunction at 3 months.

### 2.7 Statistical analysis

Continuous variables were expressed as mean ± standard deviation or median (interquartile range), according to their distribution. Categorical variables were presented as frequencies and percentages. Comparisons between groups were performed using the Student’s *t*-test or Mann–Whitney *U* test for continuous variables and the chi-square test or Fisher’s exact test for categorical variables, as appropriate.

Univariable logistic regression analysis was performed to identify potential factors associated with moderate-to-severe sensory dysfunction. Variables considered clinically relevant or showing a *p-*value <0.10 in univariable analysis were entered into the multivariable logistic regression model. Multivariable logistic regression analysis was performed to determine independent predictors of moderate-to-severe sensory dysfunction. Results were presented as odds ratios (ORs) with 95% confidence intervals (CIs).

Because balloon morphology (pear-shaped versus non–pear-shaped configuration) was considered the primary exposure variable, an exposure-based propensity score analysis was performed to reduce potential confounding caused by baseline differences between the two morphology groups. The propensity score was calculated as the probability of having a non–pear-shaped balloon configuration based on clinically relevant baseline characteristics, procedural variables, previous MVD-related factors, and surgeon-related factors. The propensity score model included demographic characteristics, disease-related variables, vascular comorbidities, physiological and biochemical parameters, trigeminal division involvement, prior microvascular decompression history, number of previous MVD procedures, time interval since the last MVD procedure (when available), balloon-related procedural parameters, surgeon identity, senior surgeon involvement, and calendar year of surgery. Surgeon-related factors and calendar year of surgery were additionally considered as potential confounders because differences in operator experience and changes in treatment practice over time may influence both balloon configuration and postoperative sensory outcomes. Surgeon identity was entered as a categorical variable, with the four operating surgeons represented as separate categories. Calendar year of surgery was modeled as a continuous variable, corresponding to a 1-year increase, whereas senior surgeon involvement was modeled as a binary categorical variable (yes vs. no). These variables were selected because they may influence both intraoperative balloon configuration and postoperative sensory outcomes.

Patients with non–pear-shaped and pear-shaped balloon configurations were matched using 1:1 nearest-neighbor propensity score matching without replacement with a caliper width of 0.2 of the standard deviation of the logit of the propensity score. Covariate balance before and after matching was assessed using standardized mean differences (SMDs), with an SMD < 0.1 considered indicative of adequate balance between groups (Figure 1).

**Figure 1:**
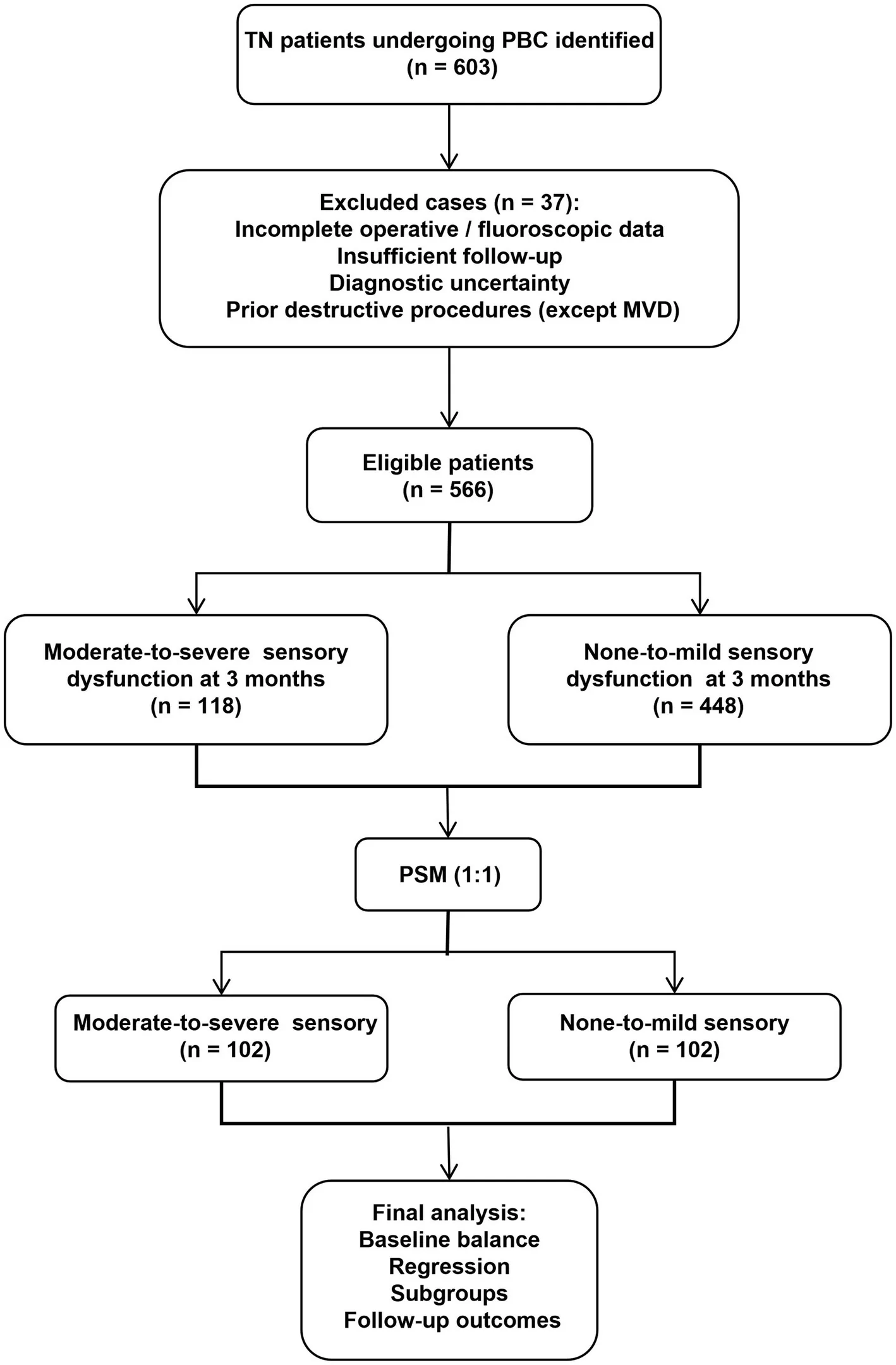
Patient selection and study flow. Flowchart summarizing case screening, exclusion, classification according to intraoperative balloon morphology, and generation of the exposure-based propensity score–matched cohort.

After propensity score matching, the association between balloon morphology and moderate-to-severe sensory dysfunction was reassessed using conditional logistic regression analysis to account for the matched cohort design. The results were expressed as ORs with 95% CIs. The robustness of the findings was further evaluated by comparing the matched cohort results with analyses performed in the full eligible cohort. As a sensitivity analysis, the association between balloon morphology and postoperative sensory dysfunction was additionally evaluated in the full eligible cohort using multivariable logistic regression adjustment. Additional multivariable logistic regression analyses were performed to further evaluate the association between balloon morphology and postoperative sensory dysfunction after adjustment for clinically relevant residual confounders, including previous MVD-related factors, surgeon identity, senior surgeon involvement, and calendar year of surgery. All statistical analyses were performed using IBM SPSS Statistics for Windows, version 27.0 (IBM Corp., Armonk, NY, USA). A two-sided *p* value <0.05 was considered statistically significant.

### 2.8 Ethical considerations

This retrospective cohort study was reviewed and approved by the Ethics Committee of the Affiliated Hospital of Beihua University (approval no. 2021–29). The requirement for informed consent for participation in this retrospective study was formally waived by the Ethics Committee because the study involved the secondary use of existing clinical records. This waiver applied exclusively to research participation and the retrospective use of clinical data and did not apply to the clinical percutaneous balloon compression (PBC) procedure itself.

All patient data were deidentified before analysis and were handled in accordance with institutional data protection and confidentiality requirements. The study was conducted in accordance with the principles of the Declaration of Helsinki.

## 3 Results

### 3.1 Patient selection and baseline characteristics

Because balloon morphology was considered the primary exposure variable, an exposure-based propensity score analysis was performed. The propensity score represented the probability of having a non–pear-shaped balloon configuration based on baseline clinical characteristics, procedural variables, previous treatment-related factors, and surgeon-related factors. An exposure-based propensity score model was constructed to estimate the probability of receiving a non–pear-shaped balloon configuration. The dependent variable for propensity score estimation was balloon morphology (non–pear-shaped versus pear-shaped configuration). Baseline demographic characteristics, disease-related factors, procedural parameters, previous treatment-related factors, and surgeon-related factors were included as covariates because these variables could influence balloon configuration selection and postoperative sensory outcomes. Postoperative sensory outcomes were not included in the propensity score model. Before propensity score matching, among the 566 included patients, pear-shaped balloon configuration was observed in 389 patients (68.7%), whereas non–pear-shaped balloon configuration was identified in 177 patients (31.3%). During the study period, all procedures were performed by four neurosurgeons experienced in percutaneous trigeminal procedures under a standardized operative protocol. Surgeon identity, senior surgeon involvement, and calendar year of surgery were considered as potential confounding variables because operator-specific practice and temporal changes in treatment patterns could influence both balloon configuration and postoperative sensory outcomes. Patients with non–pear-shaped and pear-shaped balloon configurations were subsequently matched using an exposure-based propensity score approach. A one-to-one nearest-neighbor matching procedure without replacement was applied, resulting in 102 matched pairs (204 patients) of non–pear-shaped and pear-shaped balloon configurations. After matching, baseline characteristics were well balanced between groups, with all covariates demonstrating standardized mean differences (SMDs) < 0.1 (Figure 2A). Baseline characteristics before and after propensity score matching are summarized in Tables 1, 2, respectively. The propensity score distributions of the two groups also showed substantial overlap after matching (Figure 2B), further supporting adequate covariate balance.

**Figure 2:**
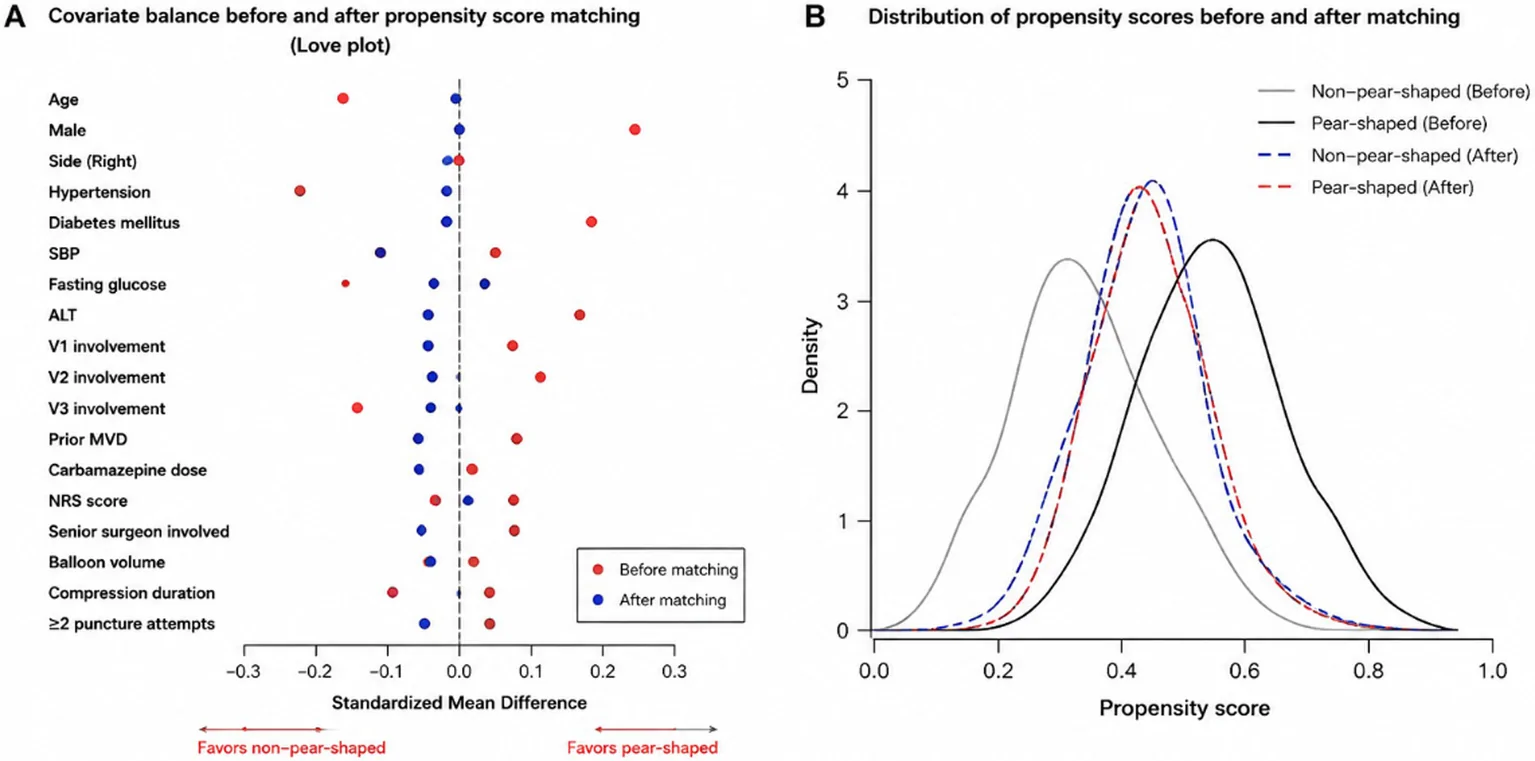
Assessment of covariate balance before and after propensity score matching according to balloon morphology. **(A)** Love plot showing standardized mean differences (SMDs) for baseline covariates before (red) and after (blue) matching. The vertical dashed line at 0 indicates perfect balance. After matching, all covariates achieved good balance with |SMD| < 0.1. **(B)** Distribution of propensity scores before and after matching for patients with non–pear-shaped and pear-shaped balloon configurations. After matching, the distributions of the two groups overlap substantially, indicating well-balanced comparison.

**Table 1:** Baseline characteristics of patients before propensity score matching according to balloon morphology.

Variables	Pear-shaped balloon (*n* = 389)	Non–pear-shaped balloon (*n* = 177)	Statistical test (value)	*p*-value
Age, years	64.1 ± 9.0	65.8 ± 8.7	*t* = −2.12	0.035
Male, *n* (%)	132 (33.9%)	55 (31.1%)	χ^2^ = 0.43	0.51
Disease duration, years	4.8 ± 2.9	5.8 ± 3.1	t = −3.75	<0.001
Primary affected division (V2/V3), *n* (%)	284 (73.0%)	124 (70.1%)	χ^2^ = 0.53	0.47
Previous MVD, *n* (%)	28 (7.2%)	16 (9.0%)	χ^2^ = 0.57	0.45
Number of previous MVD procedures, *n* (%)			χ^2^ = 0.38	0.54
1 previous MVD	25 (6.4%)	14 (7.9%)		
≥2 previous MVD procedures	3 (0.8%)	2 (1.1%)		
Time since last MVD, years	5.2 ± 2.8	5.0 ± 2.6	*t* = 0.31	0.76
Carbamazepine dose, mg/day	598 ± 184	632 ± 198	*t* = −2.02	0.044
Systolic BP, mmHg	134 ± 14	136 ± 15	*t* = −1.51	0.13
Fasting glucose, mmol/L	5.6 ± 0.9	5.8 ± 1.1	*t* = −2.12	0.035
ALT, U/L	21.8 ± 10.0	23.5 ± 11.1	*t* = −1.84	0.067
Preoperative NRS	7.9 ± 1.3	8.2 ± 1.2	*t* = −2.63	0.009
Compression duration, seconds	75 ± 12	81 ± 13	*t* = −5.43	<0.001
Balloon volume, mL	0.71 ± 0.08	0.74 ± 0.10	*t* = −3.64	<0.001
Hypertension, *n* (%)	137 (35.2%)	63 (35.6%)	χ^2^ = 0.01	0.93
Diabetes mellitus, *n* (%)	48 (12.3%)	25 (14.1%)	χ^2^ = 0.34	0.56
Senior surgeon involved, *n* (%)	310 (79.7%)	124 (70.1%)	χ^2^ = 6.24	0.012

Continuous variables are presented as mean ± standard deviation (SD), and categorical variables as number (%). MVD, microvascular decompression; BP, blood pressure; ALT, alanine aminotransferase; NRS, Numeric Rating Scale.

**Table 2:** Baseline characteristics after propensity score matching according to balloon morphology.

Variables	Pear-shaped balloon (*n* = 102)	Non–pear-shaped balloon (*n* = 102)	Statistical test (value)	*p*-value
Age, years	65.7 ± 8.9	65.9 ± 8.8	*t* = −0.16	0.87
Male, *n* (%)	34 (33.3%)	35 (34.3%)	χ^2^ = 0.02	0.88
Hypertension, *n* (%)	36 (35.3%)	37 (36.3%)	χ^2^ = 0.02	0.88
Diabetes mellitus, *n* (%)	14 (13.7%)	15 (14.7%)	χ^2^ = 0.04	0.84
Systolic BP, mmHg	135 ± 14	135 ± 15	*t* = 0.00	1.00
Fasting glucose, mmol/L	5.7 ± 1.0	5.7 ± 1.0	*t* = 0.00	1.00
V1 involvement, *n* (%)	9 (8.8%)	9 (8.8%)	χ^2^ = 0.00	1.00
V2 involvement, *n* (%)	46 (45.1%)	46 (45.1%)	χ^2^ = 0.00	1.00
V3 involvement, *n* (%)	63 (61.8%)	64 (62.7%)	χ^2^ = 0.02	0.89
Previous MVD, *n* (%)	8 (7.8%)	8 (7.8%)	χ^2^ = 0.00	1.00
Number of previous MVD procedures, *n* (%)			χ^2^ = 0.00	1.00
1 previous MVD	7 (6.9%)	7 (6.9%)		
≥2 previous MVD procedures	1 (1.0%)	1 (1.0%)		
Time since last MVD, years	5.1 ± 2.7	5.0 ± 2.6	*t* = 0.27	0.79
Disease duration, years	5.4 ± 3.2	5.5 ± 3.1	*t* = −0.22	0.83
Balloon volume, mL	0.71 ± 0.09	0.72 ± 0.09	*t* = −0.78	0.44
Compression duration, seconds	78 ± 12	79 ± 12	*t* = −0.59	0.56
Senior surgeon involved, *n* (%)	80 (78.4%)	79 (77.5%)	χ^2^ = 0.02	0.88

**V**alues are presented as mean ± standard deviation (SD) or number (%). Propensity score matching was performed according to balloon morphology, resulting in 102 matched pairs. MVD, microvascular decompression; BP, blood pressure.

### 3.2 Balloon morphology and postoperative sensory outcomes

Among the 566 patients included in the study, pear-shaped balloon configuration was observed in 389 patients (68.7%). Non–pear-shaped configurations were identified in 177 patients (31.3%), including oval-shaped balloons in 82 patients (14.5%), dumbbell-shaped balloons in 41 patients (7.2%), irregular-shaped balloons in 32 patients (5.7%), and poorly positioned balloons in 22 patients (3.9%). Representative fluoroscopic images of each morphology subtype are shown in Supplementary Figure S1. For the primary analysis, these atypical configurations were combined into a single non–pear-shaped category.

Following exposure-based propensity score matching according to balloon morphology, 204 patients were included in the comparative analysis. In the matched cohort, non–pear-shaped balloon morphology remained significantly associated with moderate-to-severe postoperative sensory dysfunction (OR, 1.86; 95% CI, 1.12–3.09; *p* = 0.017).

At 3 months, moderate-to-severe sensory dysfunction occurred in 67 patients (65.7%) with non–pear-shaped balloons compared with 52 patients (51.0%) with pear-shaped balloons (*p* = 0.034) (Figure 3A). Severity analysis showed that non–pear-shaped balloons were associated with a greater proportion of moderate and severe dysfunction (moderate: 46 patients, 45.1%; severe: 21 patients, 20.6%) compared with pear-shaped balloons (moderate: 38 patients, 37.3%; severe: 14 patients, 13.7%) (Figure 3B).

**Figure 3:**
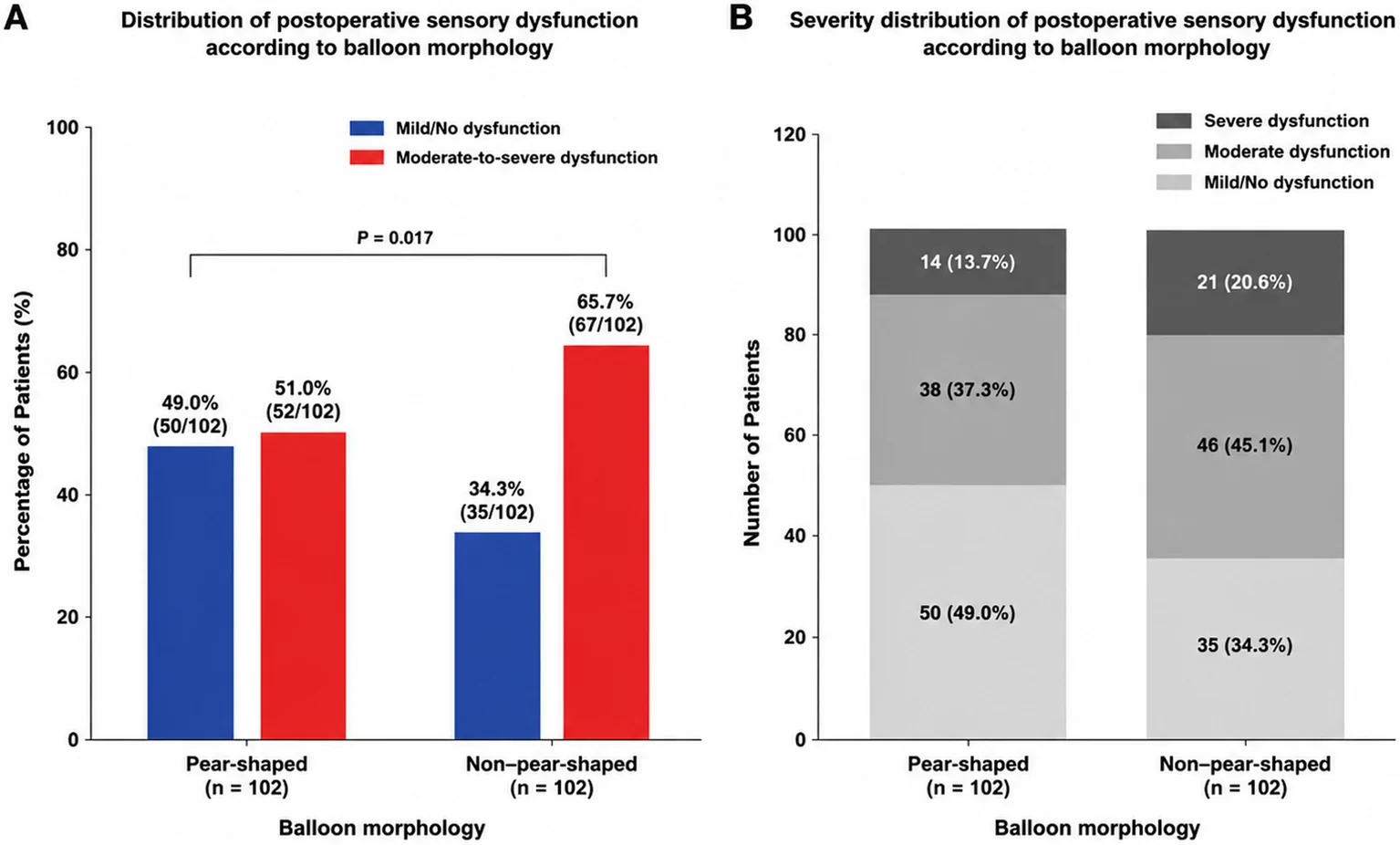
Postoperative sensory outcomes according to balloon morphology in the propensity score–matched cohort. **(A)** Distribution of postoperative sensory dysfunction (mild/no vs. moderate-to-severe) according to balloon morphology. Patients with non–pear-shaped balloon configurations had a significantly higher proportion of moderate-to-severe sensory dysfunction compared with those with pear-shaped configurations (65.7% vs. 51.0%, *p* = 0.034). **(B)** Severity of postoperative sensory dysfunction (mild/no, moderate, severe) according to balloon morphology. Non–pear-shaped balloon morphology was associated with a higher proportion of patients experiencing moderate and severe sensory dysfunction.

### 3.3 Univariable associations with postoperative sensory impairment

Univariable logistic regression was undertaken to examine the individual relationships between key clinical and procedural variables and the likelihood of developing moderate-to-severe postoperative sensory dysfunction within the matched cohort. These analyses focused on procedural and disease-related characteristics of clinical interest, together with balloon morphology as the primary exposure of interest. The results of these analyses are summarized in Table 3.

**Table 3:** Univariable logistic regression for moderate-to-severe postoperative sensory dysfunction.

Variable	OR (95% CI)	*P*-value
Balloon morphology (non–pear-shaped vs. pear-shaped)	1.98 (1.22–3.21)	0.006
Disease duration, years	1.14 (1.03–1.27)	0.012
Balloon volume, per 0.1 mL decrease	1.36 (1.09–1.72)	0.007
Compression duration, per 10 s increase	1.25 (1.02–1.57)	0.031

Values are expressed as odds ratios (ORs) with 95% confidence intervals (CIs) obtained from univariable logistic regression models. Balloon morphology was treated as a binary variable. Continuous variables were modeled on clinically meaningful scales including per year of disease duration, per 0.1 mL change in balloon volume, and per 10 s change in compression duration. All analyses were performed in the matched cohort.

Several variables demonstrated notable associations with postoperative sensory dysfunction. Individuals whose procedures resulted in a non–pear-shaped balloon configuration exhibited a higher probability of experiencing **moderate-to-severe** impairment, with an odds ratio approximately twice that observed in patients with a pear-shaped configuration. Longer disease duration before intervention also conveyed a progressively greater likelihood of postoperative dysfunction, with odds ratios exceeding unity across the observed range. Procedural factors displayed similar unadjusted trends: smaller balloon filling volumes and prolonged compression durations were each associated with increased risk, both demonstrating odds ratios above one. These associations were further evaluated in the subsequent multivariable regression analyses together with prespecified clinically relevant confounders.

### 3.4 Multivariable analysis

A multivariable logistic regression model was constructed within the balloon morphology-based propensity score-matched cohort to evaluate whether the association between balloon morphology and moderate-to-severe postoperative sensory dysfunction remained independent after additional adjustment for clinical, procedural, previous treatment-related, and surgeon-related factors. The model incorporated balloon morphology as the primary exposure variable, together with disease duration, balloon filling volume, compression duration, previous microvascular decompression (MVD) history, number of previous MVD procedures, time interval since the last MVD procedure, surgeon identity, senior surgeon involvement, and calendar year of surgery. Surgeon identity was modeled as a categorical variable, senior surgeon involvement as a binary categorical variable, and calendar year as a continuous variable. Adjusted estimates are presented in Table 4 and summarized visually in Figure 4.

**Table 4:** Multivariable analysis of moderate-to-severe postoperative sensory dysfunction in the propensity score–matched cohort.

Variable	Adjusted OR (95% CI)	*P*-value
Balloon morphology (non–pear-shaped vs. pear-shaped)	1.82 (1.09–3.05)	0.021
Disease duration, years	1.12 (1.01–1.25)	0.030
Balloon volume, per 0.1 mL decrease	1.29 (1.03–1.62)	0.028
Compression duration, per 10 s increase	1.21 (0.97–1.53)	0.086

Adjusted odds ratios (aORs) and 95% confidence intervals (CIs) were derived from the fully adjusted regression model. Balloon morphology was the primary exposure variable, with pear-shaped morphology serving as the reference category. The model additionally adjusted for disease duration, balloon filling volume, compression duration, previous MVD history, number of previous MVD procedures, time since the last MVD procedure, surgeon identity, senior surgeon involvement, and calendar year of surgery. Surgeon identity was modeled as a categorical fixed-effect variable, senior surgeon involvement as a binary categorical variable, and calendar year as a continuous variable.

**Figure 4:**
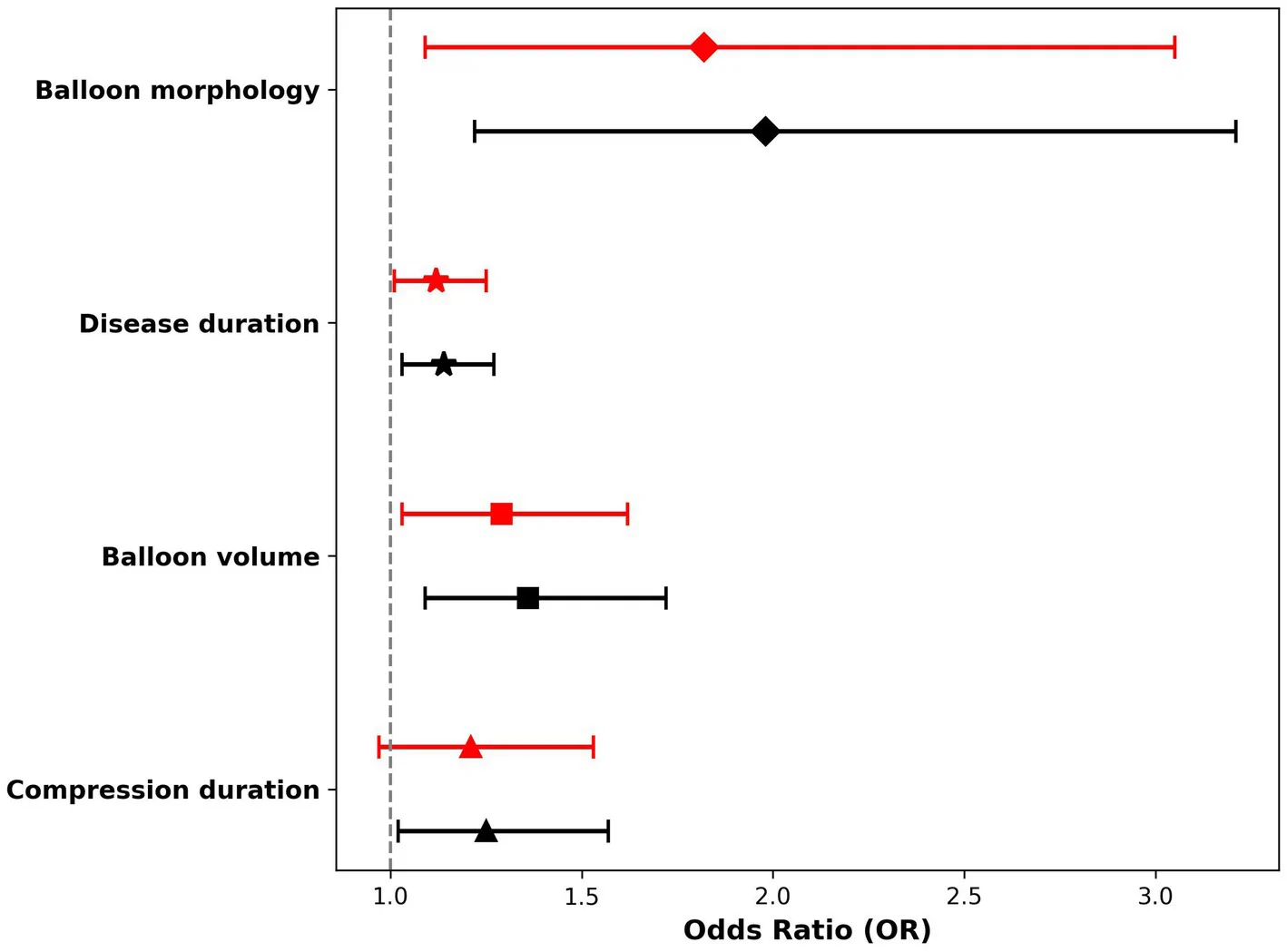
Univariable and multivariable logistic regression for moderate-to-severe postoperative sensory dysfunction. This figure illustrates the unadjusted and adjusted odds ratios for variables associated with moderate-to-severe sensory dysfunction after percutaneous balloon compression. Black markers represent univariable estimates, and red markers denote values obtained from the multivariable logistic regression model. Horizontal lines indicate 95% confidence intervals. The vertical dashed line marks an odds ratio of 1, which reflects no association. Balloon morphology served as the principal exposure, whereas disease duration, balloon filling volume, and compression duration were included as clinically relevant covariates in the multivariable model. All analyses were performed within the propensity score–matched cohort.

After simultaneous adjustment for all included variables, non–pear-shaped balloon morphology remained independently associated with a higher likelihood of moderate-to-severe postoperative sensory dysfunction, with an adjusted odds ratio of 1.82 (95% CI, 1.09–3.05; *p* = 0.021). Longer preoperative disease duration retained a modest but measurable contribution to increased risk on a per-year basis. Balloon volume demonstrated a similar direction of effect, such that smaller filling volumes were associated with a higher probability of postoperative sensory impairment. Compression duration showed a positive but less consistent association after adjustment.

Previous MVD history, number of prior MVD procedures, time since the last MVD procedure, surgeon identity, senior surgeon involvement, and calendar year of surgery were additionally evaluated as potential confounding factors; however, none demonstrated a stronger independent association than balloon morphology after adjustment. The inclusion of these variables did not materially alter the association between non–pear-shaped balloon configuration and postoperative sensory dysfunction.

Collectively, these findings indicate that non–pear-shaped balloon morphology represents an independent predictor of moderate-to-severe postoperative sensory dysfunction after accounting for disease-related characteristics, procedural parameters, previous surgical history, surgeon-related factors, and calendar year of surgery.

### 3.5 Postoperative evolution of sensory dysfunction across follow-up

Routine follow-up assessments were available for all patients in the balloon morphology-based propensity score-matched cohort at 1, 3, and 12 months, allowing evaluation of postoperative sensory recovery according to balloon configuration. A consistent temporal pattern was observed across follow-up assessments. At the one-month evaluation, moderate-to-severe sensory dysfunction was observed more frequently among patients with non–pear-shaped balloon configurations than among those with pear-shaped balloons (70.6% vs. 49.0%, respectively; *p* = 0.002) (Table 5). Although both groups demonstrated gradual sensory improvement over time, the difference between balloon morphologies remained evident at the three-month primary outcome assessment. At 3 months, moderate-to-severe sensory dysfunction persisted in 67 patients (65.7%) with non–pear-shaped balloons compared with 52 patients (51.0%) with pear-shaped balloons (*p* = 0.034).

**Table 5:** Postoperative sensory dysfunction according to balloon morphology at 1, 3, and 12 months after propensity score matching.

Follow-up time	Sensory category	Pear-shaped balloon (*n* = 102)	Non–pear-shaped balloon (*n* = 102)	*P*-value
1 month	Mild/No	52 (51.0%)	30 (29.4%)	0.002
	Moderate/Severe	50 (49.0%)	72 (70.6%)	
3 months	Mild/No	50 (49.0%)	35 (34.3%)	0.034
	Moderate/Severe	52 (51.0%)	67 (65.7%)	
12 months	Mild/No	72 (70.6%)	55 (53.9%)	0.017
	Moderate/Severe	30 (29.4%)	47 (46.1%)	

Further recovery was observed at the 12-month follow-up. The proportion of patients with moderate-to-severe sensory dysfunction decreased in both groups; however, it remained higher among patients with non–pear-shaped balloon configurations compared with those with pear-shaped balloons (46.1% vs. 29.4%, respectively; *p* = 0.017). These longitudinal findings are illustrated in Figure 5, demonstrating progressive improvement in sensory outcomes after percutaneous balloon compression while showing a consistently higher burden of persistent sensory dysfunction among patients with non–pear-shaped balloon morphology. The observed differences across follow-up time points suggest that intraoperative balloon configuration may influence both early postoperative sensory changes and long-term sensory recovery patterns.

**Figure 5:**
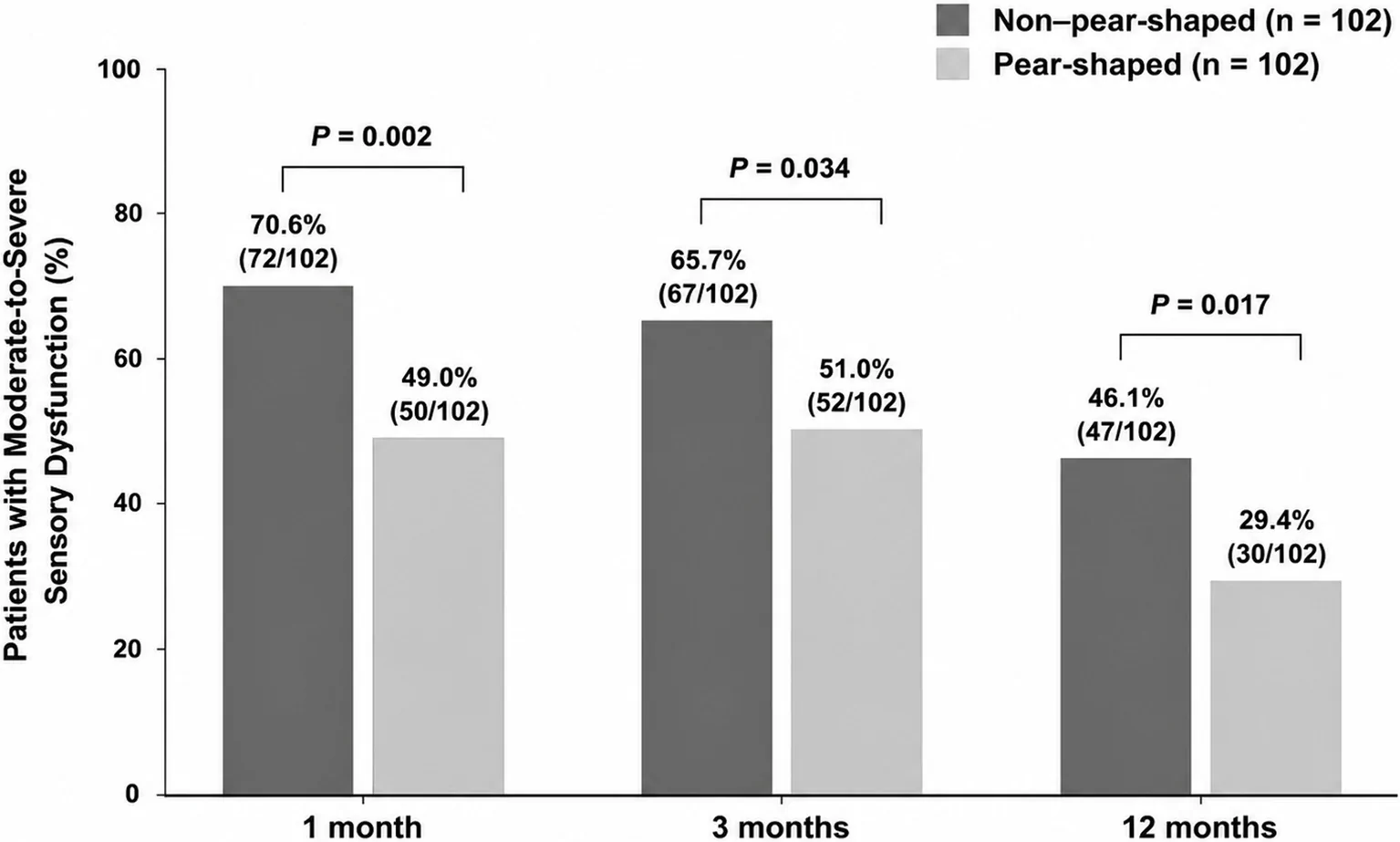
Temporal evolution of moderate-to-severe sensory dysfunction according to balloon morphology in the propensity score–matched cohort. Bars indicate the proportion (%) of patients with moderate-to-severe sensory dysfunction (BNI grades III–V) at each follow-up time point. Comparisons between groups at each time point were performed using the chi-square test.

### 3.6 Severe sensory complications

Severe sensory complications were additionally assessed separately. Among all included patients, painful dysesthesia occurred in 18 patients (3.2%), corneal sensory loss occurred in 7 patients (1.2%), and anesthesia dolorosa was observed in 3 patients (0.5%). The incidence of these severe sensory complications was compared between the pear-shaped and non–pear-shaped balloon configuration groups. After propensity score matching, painful dysesthesia occurred in 3 patients (2.9%) in the pear-shaped balloon group and 4 patients (3.9%) in the non–pear-shaped balloon group (*p* = 0.70); corneal sensory loss occurred in 1 patient (1.0%) and 2 patients (2.0%), respectively (*p* = 0.56); and anesthesia dolorosa occurred in 1 patient (1.0%) and 1 patient (1.0%), respectively (*p* > 0.99). No significant differences were observed between groups.

### 3.7 Subgroup and sensitivity analyses

To evaluate whether the association between balloon morphology and postoperative sensory outcomes remained consistent across clinically relevant subgroups, predefined subgroup analyses were performed within the balloon morphology-based propensity score-matched cohort. Patients were stratified according to disease duration, balloon filling volume, and compression duration, which were selected as clinically relevant factors for assessment of potential effect modification. Logistic regression models were applied within each subgroup to estimate the association between non–pear-shaped balloon morphology (versus pear-shaped morphology) and the risk of moderate-to-severe sensory dysfunction.

Across all examined subgroups, non–pear-shaped balloon configuration consistently demonstrated a higher likelihood of moderate-to-severe sensory dysfunction compared with pear-shaped balloon configuration (Table 6). Although the magnitude of association varied among different clinical and procedural strata, the direction of effect remained consistent. The estimated odds ratios ranged from approximately 1.6 to 2.9, indicating a persistent association between non–pear-shaped balloon morphology and unfavorable sensory outcomes.

**Table 6:** Subgroup analysis of the association between non–pear-shaped balloon morphology and moderate-to-severe sensory dysfunction.

Subgroup	Pear-shaped balloon n/N (%)	Non–pear-shaped balloon n/N (%)	OR (95% CI)	*P* for interaction
Disease duration <3 years	18/54 (33.3%)	28/46 (60.9%)	2.94 (1.33–6.48)	0.18
Disease duration ≥3 years	34/48 (70.8%)	39/56 (69.6%)	2.01 (0.91–4.45)	
Balloon volume <0.70 mL	22/48 (45.8%)	30/48 (62.5%)	2.10 (0.91–4.86)	0.27
Balloon volume ≥0.70 mL	30/54 (55.6%)	37/54 (68.5%)	1.74 (0.74–4.10)	
Compression duration <80 s	19/55 (34.5%)	32/55 (58.2%)	2.65 (1.16–6.07)	0.31

The subgroup associations are presented graphically in Figure 6. No significant interaction was detected between balloon morphology and disease duration, balloon volume, or compression duration (all *P* for interaction >0.05), suggesting that the effect of balloon configuration on sensory outcomes was generally consistent across these clinical and procedural characteristics. To assess the robustness of the findings beyond the propensity score-matched cohort, a sensitivity analysis was performed using the full eligible cohort of 566 patients. Multivariable logistic regression was applied with adjustment for clinically relevant confounders, including disease duration, balloon filling volume, compression duration, previous MVD history, number of previous MVD procedures, time interval since the last MVD procedure, surgeon identity, senior surgeon involvement, and calendar year of surgery. Surgeon identity was modeled as a categorical variable, senior surgeon involvement as a binary categorical variable, and calendar year as a continuous variable. After adjustment, non–pear-shaped balloon morphology remained independently associated with moderate-to-severe postoperative sensory dysfunction (adjusted OR, 1.79; 95% CI, 1.24–2.58; *p* = 0.002). These findings were consistent with those obtained from the exposure-based propensity score-matched cohort, supporting the robustness of the observed association between non–pear-shaped balloon configuration and postoperative sensory dysfunction.

**Figure 6:**
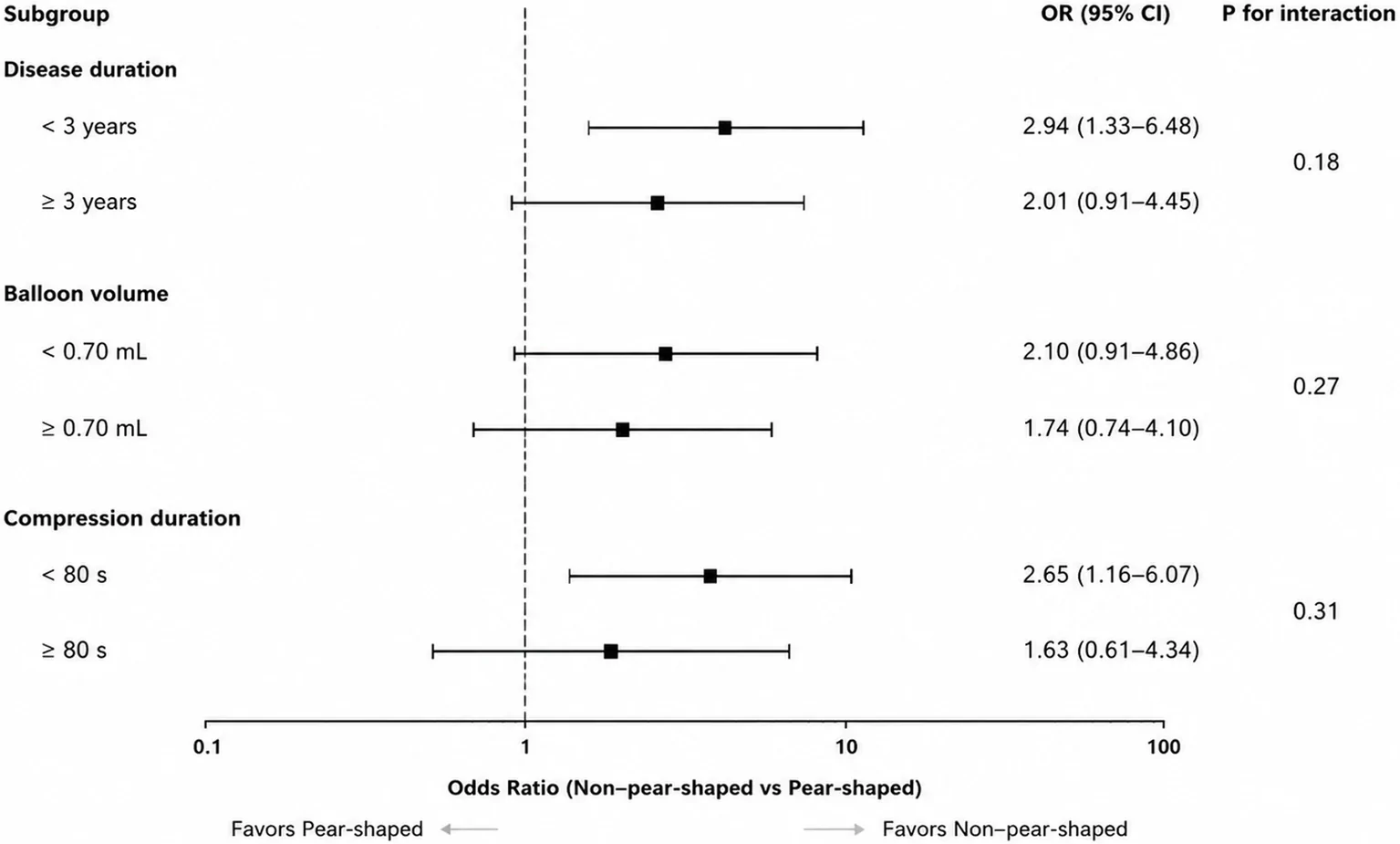
Subgroup analysis of the association between non–pear-shaped balloon morphology and moderate-to-severe sensory dysfunction at 3 months. Odds ratios (*OR*) compare non–pear-shaped versus pear-shaped balloon morphology for moderate-to-severe sensory dysfunction (yes vs. no) at 3 months within each subgroup. *OR*s were estimated using logistic regression within each stratum. *P* for interaction tests whether the effect of balloon morphology differs across subgroups. *CI*, confidence interval.

Overall, the subgroup and sensitivity analyses support the robustness of the primary finding that non–pear-shaped balloon morphology is independently associated with an increased risk of moderate-to-severe postoperative sensory dysfunction after percutaneous balloon compression.

## 4 Discussion

This study examines a large cohort of patients treated with percutaneous balloon compression for trigeminal neuralgia and identifies a consistent relationship between intraoperative balloon morphology and postoperative sensory outcomes. Within a propensity score–matched population, non–pear-shaped configurations were associated with a higher probability of moderate-to-severe numbness at the three-month evaluation. This association persisted after adjustment for disease duration, balloon volume, and compression time and remained stable across clinically relevant subgroups. These findings suggest that balloon morphology, as observed on intraoperative fluoroscopy, provides useful information about procedural configuration and may serve as a clinical marker associated with sensory recovery.

The observed pattern should be interpreted in the context of established procedural anatomy and fiber selectivity. Although the operation was historically described as compression of the gasserian ganglion, current anatomical understanding suggests that standard balloon inflation does not necessarily injure the ganglion itself (11). Instead, percutaneous balloon compression is believed to preferentially affect large myelinated trigeminal fibers rather than causing generalized injury to all sensory fibers (27, 28). The fluoroscopic morphology likely reflects the relationship of the balloon to the trigeminal cistern, Meckel’s cave, and the porus trigeminus region (29, 30). A pear-shaped configuration has traditionally been regarded as a favorable fluoroscopic appearance, although it does not necessarily indicate a specific pattern of neural compression (31). Non–pear-shaped morphology may reflect anatomical variation, catheter position, or differences in balloon expansion (19). Therefore, these findings should be interpreted as an association between balloon configuration and sensory outcome.

The finding that smaller balloon volume was associated with greater sensory dysfunction also requires careful interpretation. Accordingly, the present study cannot determine whether differences in sensory outcome were related to balloon pressure, balloon position, balloon shape, or a combination of procedural factors. One possible explanation is that smaller filling volumes associated with sensory dysfunction may reflect balloon positioning that involves the proximal porus trigeminus region, where retroganglionic large myelinated fibers may be more vulnerable (32). However, this remains a hypothesis and cannot be confirmed from the present retrospective imaging data. Future studies incorporating intraluminal pressure monitoring, more detailed morphometric imaging, and representative examples of cisternal and porus-region balloon configurations would help clarify this relationship.

The findings also underscore the importance of sensory outcomes as determinants of overall procedural success. Although percutaneous balloon compression is valued for rapid analgesic effect and a relatively low risk of serious complications, persistent numbness remains a concern for many patients (33–35). The modified Barrow Neurological Institute classification allowed structured characterization of sensory changes and demonstrated a clear gradient in outcomes across balloon morphology categories. Longitudinal analyses showed that sensory disturbances tended to improve over time in both morphology groups, yet the difference between pear-shaped and non–pear-shaped configurations persisted across follow-up. These patterns suggest that intraoperative morphology may provide an early signal associated with subsequent sensory trajectory.

Several practical implications emerge from this work. Because intraoperative fluoroscopy is widely available in most tertiary hospitals, the morphological assessment we used does not require specialized technology and can be readily implemented in routine practice. Balloon morphology is immediately observable during the procedure and may help surgeons recognize suboptimal configuration or positioning. However, morphology-based decisions should be made cautiously because balloon shape is an indirect marker and does not directly measure balloon pressure, neural compression, selective fiber injury, or ganglion injury. In particular, because intraluminal pressure was not recorded, morphology should not be interpreted as a surrogate for the mechanical pressure applied during balloon inflation. Incorporating morphology assessment with other procedural information, including catheter position, balloon volume, compression duration, and ideally intraluminal pressure in future studies, may improve the consistency and predictability of surgical outcomes.

This study has limitations that warrant consideration. Patients without complete follow-up were excluded, which may introduce selection bias and could preferentially omit individuals with unfavorable or atypical postoperative trajectories. The retrospective, single-center design may restrict generalizability, although the consistency of findings across subgroups suggests that the observed associations are not attributable solely to institutional practice patterns. Sensory evaluations relied on clinical examination and patient report rather than quantitative neurophysiological testing, although the use of a standardized grading scale mitigates subjectivity. Balloon morphology was categorized qualitatively, and although interobserver agreement was high, more granular morphometric analysis might provide additional insight. In addition, fluoroscopic images were not systematically collected to distinguish purely cisternal compression from configurations involving the porus trigeminus region. Furthermore, the binary classification of balloon morphology into “pear” and “non-pear” oversimplifies a spectrum of fluoroscopic shapes, some of which (e.g., pill-shaped vs. pawn-shaped) may reflect distinct anatomical positions and degrees of neural compression. Although non–pear-shaped configurations were further classified into oval-shaped, dumbbell-shaped, irregular-shaped, and poorly positioned subtypes, these subgroups were combined for the primary analysis because of sample size limitations. Further prospective or multicenter studies incorporating standardized imaging, pressure monitoring, and detailed anatomical classification are needed.

An additional limitation is that this study focused primarily on postoperative sensory dysfunction and did not evaluate whether balloon morphology was associated with differences in pain relief durability or recurrence rates. Therefore, although non–pear-shaped balloon configurations were associated with increased sensory morbidity, the present study cannot determine whether these configurations provide potential advantages in terms of long-term pain control. Future studies incorporating both analgesic outcomes and sensory complications are required to define the overall clinical benefit associated with different balloon morphologies.

Balloon volume and compression duration are important procedural determinants of sensory outcomes and may partially explain the observed association between balloon morphology and postoperative sensory dysfunction. Although these variables were adjusted for in multivariable analyses, their independent contributions cannot be completely separated in this retrospective design.

Furthermore, previous treatment history and surgeon-related factors may represent additional sources of residual confounding. Previous MVD history, number of previous MVD procedures, time interval since the last MVD procedure, surgeon identity, senior surgeon involvement, and calendar year of surgery were therefore considered and incorporated into the adjusted analyses. However, residual confounding related to unmeasured technical aspects of previous surgery, subtle differences in operative technique, or other operator-dependent factors cannot be completely excluded because of the retrospective nature of the study. Although the propensity score analysis was performed using an exposure-based framework according to balloon morphology and adjustment for measured covariates was extensive, the possibility of residual or unmeasured confounding remains. Future prospective studies with standardized assessment of procedural factors and detailed intraoperative parameters are warranted to further validate the relationship between balloon morphology and postoperative sensory outcomes.

In summary, balloon morphology observed during percutaneous balloon compression serves as a fluoroscopic marker of procedural configuration rather than a direct measure of trigeminal ganglion deformation, generalized sensory fiber injury, or balloon pressure. Non–pear-shaped configurations were associated with a greater likelihood of moderate-to-severe postoperative sensory dysfunction, and this association was consistent across procedural and clinical subgroups (36). These findings support the clinical value of intraoperative fluoroscopic morphology while emphasizing the need for cautious mechanistic interpretation and further prospective anatomical, pressure-based, and fiber-specific studies with complete and accessible reference documentation.

## Data Availability

The raw data supporting the conclusions of this article will be made available by the authors, without undue reservation.

## Supplementary material

SUPPLEMENTARY FIGURE S1 Representative fluoroscopic images of balloon morphology configurations during percutaneous balloon compression. Images were obtained at maximal balloon inflation after complete contrast filling using biplanar fluoroscopy. **(A)** Pear-shaped: the balloon shows a characteristic waist at the level of Meckel’s cave on both lateral and anteroposterior (AP) views. **(B)** Oval-shaped: the balloon demonstrates symmetric expansion without a distinct waist. **(C)** Dumbbell-shaped: the balloon shows two-lobed expansion with a central narrowing. **(D)** Irregular-shaped: the balloon demonstrates an asymmetric contour with uneven expansion. **(E)** Poorly positioned: the balloon does not appropriately occupy the intended compression region of Meckel’s cave. Balloon morphology was evaluated using fluoroscopic images obtained at maximal balloon inflation after complete contrast filling. Images were acquired using biplanar fluoroscopy, including anteroposterior (AP) and lateral projections.
